# Inhibition of NLRP3 Inflammasome Ameliorates Cerebral Ischemia-Reperfusion Injury in Diabetic Mice

**DOI:** 10.1155/2018/9163521

**Published:** 2018-04-24

**Authors:** Pu Hong, Feng-Xian Li, Ruo-Nan Gu, Ying-Ying Fang, Lu-Ying Lai, Yong-Wei Wang, Tao Tao, Shi-Yuan Xu, Zhi-Jian You, Hong-Fei Zhang

**Affiliations:** ^1^Department of Anesthesiology, Zhujiang Hospital of Southern Medical University, Guangzhou, China; ^2^Department of Anesthesiology, The Second Affiliated Hospital, Shantou University Medical College, Shantou, Guangdong, China; ^3^Department of Neurobiology, Southern Medical University, Guangzhou, China; ^4^Department of Anesthesiology, Nanfang Hospital, Southern Medical University, Guangzhou, China

## Abstract

Sustained activation of NLRP3 inflammasome is closely related to diabetes and stroke. However, it is unknown whether NLRP3 inflammasome plays an essential role in stroke in diabetes. We aim to investigate the effect and the potential mechanism of NLRP3 inflammasome in diabetic mice with cerebral ischemia-reperfusion injury. A type 2 diabetic mouse model was induced by a high-fat diet and streptozotocin (STZ). Diabetic mice received MCC950 (the specific molecule NLRP3 inhibitor) or vehicle 60 minutes before the middle cerebral artery occlusion (MCAO) and reperfusion. MCC950 reduced the neurological deficit score of 24 h after cerebral ischemia reperfusion and improved the 28-day survival rate of cerebral ischemia-reperfusion injury in diabetic mice. Furthermore, we found that the mRNA transcription levels of NLRP3, IL-1*β*, and caspase-1 in the core ischemic area were remarkably amplified in diabetic mice with cerebral ischemia-reperfusion injury, whereas this phenomenon was obviously attenuated by MCC950 pretreatment. In conclusion, the NLRP3 inflammasome was involved in the complex diseases of diabetic stroke. MCC950, the NLRP3 specific inhibitor, ameliorated diabetic mice with cerebral ischemia-reperfusion injury and improved the 28-day survival rate during the recovery stage of ischemic stroke.

## 1. Introduction

Stroke is threatening human health every day worldwide. The number of stroke patients is about 30 million annually, mainly for ischemic stroke. The key to the treatment of cerebral infarction is to restore the cerebral blood flow perfusion in the ischemic area. However, cerebral blood flow reperfusion causes damage or dysfunction of some ischemic brain tissue, that is, cerebral ischemia-reperfusion injury. At present, thrombolytic therapy is one of the most effective treatments for ischemic stroke; however, the therapeutic window is narrow within the initial 3 to 5 hours. Diabetes aggravates the fragility and vulnerability of cerebral vessels, which makes it an important risk for ischemic stroke. Evidences showed that the incidence of diabetes mellitus combined with stroke is around 14.18% [1–3]. Diabetes and ischemic stroke are both common diseases in the clinic with the limited means of prevention and treatment. Therefore, it is of great practical significance to explore the pathogenesis and effective prevention and treatment.

A variety of major hazards to human health such as type 2 diabetes and diseases of the nervous system have been detected in chronic inflammation in evidence, but the specific molecular and cellular mechanisms of inflammation in the pathogenesis of those diseases are not entirely clear. An effective anti-inflammatory target has not been identified. NLRP3 inflammasome, one of the NOD-like receptors, is a multiprotein complex in cells, with three domains of NLRP3, caspase-1, and ASC as the core proteins. Its main function is to identify the risk signals of exogenous infection and internal injury of various pathogenic microorganisms and mediate the activation of caspase-1 and process pro-IL-*β* into a mature active form to secrete extracellularly, so as to activate a natural immune response, resulting in multicascade inflammatory response and cell apoptosis [4]. NLRP3 inflammasome has proved to be involved in the development of many diseases, like stroke, type 2 diabetes, Alzheimer's disease, etc. [5–7]. Our previous studies deeply revealed the close relationship between inflammasome and pain [8]. An inflammatory cascade of diabetic cerebral ischemia induces a series of complex changes in intracellular homeostasis, and NLRP3 inflammasome is an important factor in inflammatory after stroke. However, there is no study on whether it is the leading factor in the poststroke injury of diabetes mellitus. The key mechanism of NLRP3 inflammasome and diabetic ischemic stroke remains unclear.

Based on these backgrounds, in the current study, by establishing the mouse model of type 2 diabetes and middle cerebral artery occlusion (MCAO), we initially investigated whether inhibition of activation of NLRP3 inflammasome reduces cerebral ischemia-reperfusion injury in diabetic mice. As one of the most important proinflammatory factors, IL-1*β* aggravates the progress of cerebral ischemia-reperfusion injury and type 2 diabetes. Therefore, testing IL-1*β*/caspase-1 expression may indicate whether inhibition of NLRP3 inflammasome is essential for DM mice with cerebral I/R injury. And more importantly, we try to find a new approach for the treatment of diabetes combined with ischemic stroke to improve long-term survival.

## 2. Materials and Methods

### 2.1. Animal

The study protocols were approved by the Medical Faculty Ethics Committee of Southern Medical University. All experimental procedures were performed in accordance with the National Institutes of Health Guide for the Care and Use of Laboratory Animals. Male mice (C57BL/6J, 14–18 g), 4–6 weeks old, were obtained from the Animal Experimental Center of Southern Medical University (SCXK 2016-0041, Guangdong, China) and housed under pathogen-free conditions with a 12 hr light/dark cycle and were provided ad libitum tap water and food before the experiment.

### 2.2. Type 2 Diabetes Mouse Model

**Figure 1:**
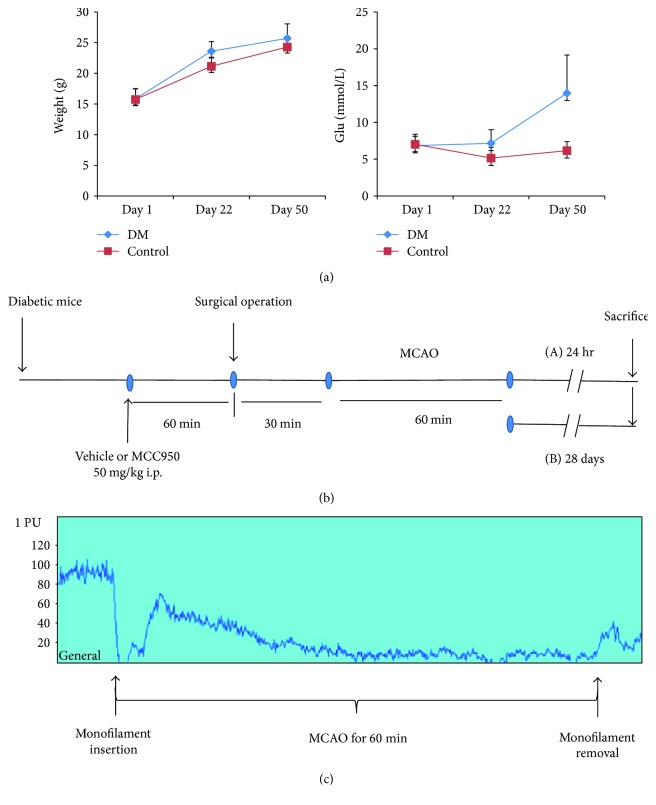
Experimental procedure used in the study and changes in cerebral blood flow (CBF) in animals subjected to MCAO by a laser Doppler. (a) Trend in blood glucose and weight of mice injected intraperitoneally with STZ or vehicle (PBS) on day 22. (b) In the first experiment (A), diabetic mice were assessed with the neurologic scores and infarct size 24 hours after MCAO. In the second experiment (B), the survival of diabetic mice was observed until twenty-eight days after MCAO. (c) Laser Doppler measurements revealed that the middle cerebral artery occlusion reduced CBF sharply when the monofilament was inserted. Then cerebral blood flow showed compensatory increase and then maintained the hypoperfusion. After reperfusion, CBF could not restore to the baseline in the ischemia core for a short period of time.

As previous report [9], a diabetic mouse model was generated by feeding a high-fat diet for 3 weeks, followed by streptozotocin (STZ, Sigma, USA) intraperitoneal injection at a dose of 100 mg/kg (dissolved in 0.1 mol/L sodium citrate solution to a final concentration of 10 g/L pH adjusted to 4.2–4.3). The high-fat diet was preceded for 4 weeks before the MCAO surgery. On day 50, all mice fast for 6 to 8 hr and their blood glucose levels from tails were analyzed using a blood glucose monitoring system (Abbott Leeshai, China). Taken the fasting, blood glucose ≥ 10 mmol/L was considered the success of diabetic mouse model establishment. For concerning the aging problem, we have set up the control group which was fed a normal diet and was injected with vehicle for STZ and was raised in the same environment. A trend in blood glucose and weight of both groups of mice is shown in Figure 1(a). Mice that died less than 28 days after STZ or vehicle injection were excluded.

### 2.3. Treatment and Grouping

Diabetic mice were randomly divided into 4 groups: sham/MCC950 group, sham/vehicle group, MCAO/MCC950 group, and MCAO/vehicle group. MCC950 was dissolved in 0.1 M PBS (pH 7.4) to a final concentration of 5 mg/mL. According to a previous report [10] on the pharmacokinetics of MCC950 in vitro and in vivo, 50 mg/kg MCC950 (HY-12815A, MCE, USA) intraperitoneal injection was administrated 60 min before MCAO or sham operation for the pretreatment group. Injecting an equal volume of 0.1 M PBS (pH 7.4) into the vehicle group mice 60 min before MCAO or sham operation.

### 2.4. Focal Cerebral Ischemia and Neurologic Evaluation

The experimental procedure shown in Figure 1(b) followed the protocol of our previous study [11]. Moreover, we also monitor the regional cerebral blood flow to confirm the location of the nylon monofilament with a laser Doppler prone (Figure 1(c)). MCAO procedure was adapted as our previous study [11]. Briefly, a transient occlusion of artery flow was established by inserting a 4-0 nylon monofilament (0.23–0.25 mm in diameter, Yushun Bio Technology Co. Ltd., China) into the right external carotid artery and advancing it to occlude the middle cerebral artery for 60 minutes before the filament was withdrawn. Mice spontaneously breathed 2% isoflurane (RWD Life Science Co. Ltd., China) driven by oxygen-enriched air (fraction of inspired oxygen: 40%) through a facemask. Sham-operated mice underwent an identical procedure without monofilament insertion. During the whole procedure, the rectal temperature of the mice was kept at 37 ± 0.5°C with a heating pad. The mouse that died due to anesthesia and surgical operation was excluded.

The neurologic deficit score was examined 24 hours after MCAO by a researcher who was blinded to the treatment. Scores were made according to a neurologic grading scale [12]: grade 0, no signs of neurologic deficit; grade 1, mild injury, unable to extend the contralateral forelimb completely by the tail; grade 2, moderate injury, circling to the contralateral side while walking; grade 3, severe focal injury, dumping to the opposite side at rest; and grade 4, severe damage and no spontaneous activity with conscious disturbance. A mouse was excluded if it scored 0 or 4 or died within 24 hours after the procedure.

### 2.5. Cerebral Infarction Volume Evaluation

Mice were euthanized 24 hr after MCAO reperfusion. Brains were removed and sectioned into coronal slices by a matrix for a mouse brain. The infarct size was measured by 2% 2,3,5-triphenyltetrazolium chloride (TTC) staining. Infarction volume was investigated by a blinded observer by ImageJ software (version 1.61; National Institutes of Health, Bethesda, MD).

### 2.6. Survival Analysis

The vital signs of mice were observed every 12 h after MCAO reperfusion. The time of death was recorded. And data were censored on the 28th day after surgery.

### 2.7. Real-Time Quantitative RT-PCR

The expression of NLRP3, IL-1*β*, and caspase-1 mRNAs was determined by real-time quantitative reverse transcription polymerase chain reaction (RT-PCR). Briefly, total RNA was isolated from the ischemia core area by the TRIzol Reagent (Invitrogen, USA) and cDNA was synthesized by the GeneAmp PCR System 9700 (ABI, USA). Quantitative real-time PCR was performed by the SYBR Green kit (Takara, Japan) on a LightCycler480 System (Roche Diagnostics). The reaction was performed with a predegeneration step at 95°C for 30 s and annealing and extension at 60°C for 20 s for 40 cycles. The relative quantity of the target mRNA was normalized to the level of *β*-actin mRNA as the internal control.  Table 1 describes the sequences of gene-specific primers used in this study. Primer sequences of targeted gene were used as previously described [13]. The relative fold change of NLRP3, IL-1*β*, and caspase-1 mRNAs was determined by the 2^−ΔΔCt^ method.

### 2.8. Statistical Analysis

The data were presented as the mean ± standard deviation (SD). The means were compared by a two-tailed unpaired *t*-test, one-way analysis of variance (ANOVA) the for comparison of multiple samples, and Log-rank test for the statistical analysis of survival with Prism5 software (GraphPad, CA, USA). A *P* value < 0.05 was considered statistically significant.

## 3. Results

### 3.1. MCC950 Downregulated the NLRP3, IL-1β, and Caspase-1 in the Core Area of Cerebral Ischemia

**Figure 2:**
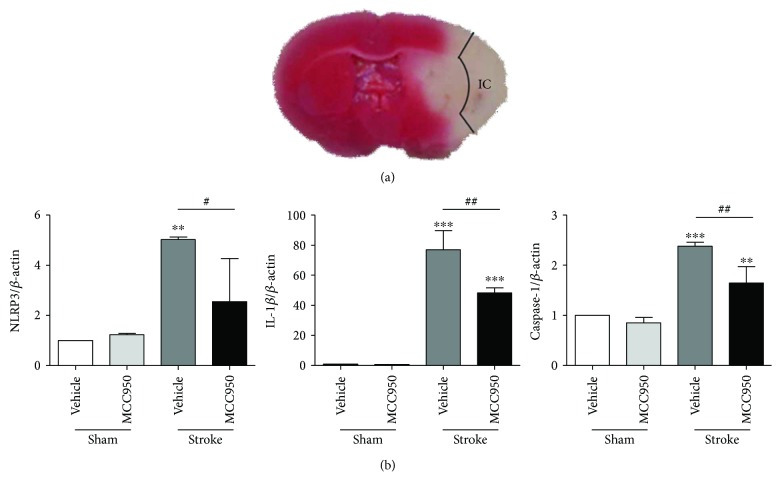
MCC950 downregulated the NLRP3, IL-1*β*, and caspase-1 in the core area of cerebral ischemia. (a) Whole brain section stained with 2% TTC stain. The area in which cortical brain tissues from the boundary zone adjacent to the ischemic core (IC) in each group were dissected on ice and mRNA was extracted. (b) The bar graphs represented, respectively, the relative gene expression changes of NLRP3, IL-1*β*, and caspase-1. *β*-Actin housekeeping was used as an endogenous reference gene. Compared with the sham/vehicle group, the NLRP3, IL-1*β*, and caspase-1 mRNA expression in the ischemic core area of the stroke/vehicle group significantly increased (^∗∗^*P* < 0.01, ^∗∗∗^*P* < 0.001, and ^∗∗∗^*P* < 0.001, resp., 3 mice per group). 24 hours after MCAO in diabetic mice, MCC950 significantly inhibited the mRNA expression of NLRP3, IL-1*β*, and caspase-1 in the core area of cerebral ischemia compared with the vehicle group (^#^*P* < 0.05, ^##^*P* < 0.01, and ^##^*P* < 0.01, resp., 3 mice per group). ^∗∗^*P* < 0.01 and ^∗∗∗^*P* < 0.001 compared with the sham group; ^#^*P* < 0.05 and ^##^*P* < 0.01 between the 2 indicated groups.

To provide evidence of MCC950 to the protective effect of cerebral ischemia reperfusion in diabetic mice, we measured the mRNA levels of NLRP3, IL-1*β*, and caspase-1 in the boundary zone adjacent to the ischemic core. Compared to the sham/vehicle group, the mRNA expression of NLRP3, IL-1*β*, and caspase-1 was obviously increased in the ischemic core area of the stroke/vehicle group (1.000 ± 0.000 versus 5.024 ± 0.099 for NLRP3, 1.000 ± 0.000 versus 77.070 ± 12.670 for IL-1*β*, and 1.000 ± 0.000 versus 2.381 ± 0.078 for caspase-1; *P* < 0.01, *P* < 0.001, and *P* < 0.001, resp.; 3 mice per group) (Figure 2(b)). As expected, MCC950 significantly inhibited the mRNA transcription levels of NLRP3, IL-1*β*, and caspase-1 in the ischemic core area of the brain 24 hr after MCAO reperfusion (5.024 ± 0.099 versus 2.551 ± 1.711 for NLRP3, 77.070 ± 12.670 versus 48.330 ± 3.370 for IL-1*β*, and 2.381 ± 0.078 versus 1.645 ± 0.329 for caspase-1; *P* < 0.05, *P* < 0.01, and *P* < 0.01, resp.; 3 mice per group) (Figure 2(b)).

### 3.2. NLRP3 Inhibition Reduced the Neurological Deficit Score in Diabetic Mice

**Figure 3:**
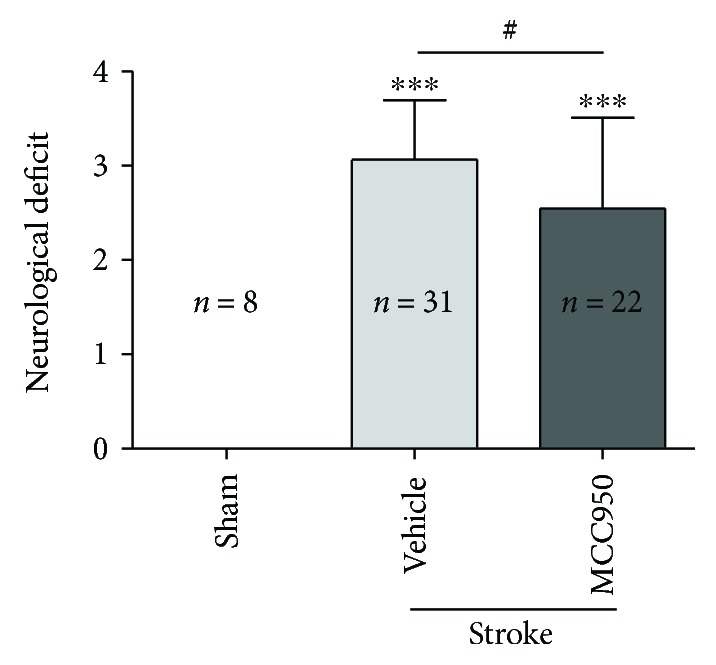
NLRP3 inhibition reduced the neurological deficit score in diabetic mice. The neurologic score was assessed 24 hours after MCAO. MCC950 reduces the neurological deficit score in diabetic mice. The neurological deficit scores in the stroke/vehicle groups were significantly higher than those in the sham/vehicle groups (^∗∗∗^*P* < 0.001). The neurologic scores were significantly ameliorated via 50 mg/kg of MCC950 i.p. pre-MCAO compared with the vehicle (^#^*P* < 0.05). ^∗∗∗^*P* < 0.001 compared with the sham group. ^#^*P* < 0.05 between the 2 indicated groups.

The neurological deficit scores in the stroke/vehicle groups were significantly higher than those in the sham/vehicle groups (3.065 ± 0.629 versus 0.000 ± 0.000, *P* < 0.001). When compared with the vehicle group, MCC950, the NLRP3 inhibitor significantly reduced the neurological deficit score 24 hr after cerebral ischemia reperfusion in diabetic mice (2.545 ± 0.963 versus 3.065 ± 0.629, *P* < 0.05, *n* = 22 and 31) (Figure 3).

### 3.3. NLRP3 Inhibition Enhanced the 28-Day Survival Rate in Diabetic Mice

**Figure 4:**
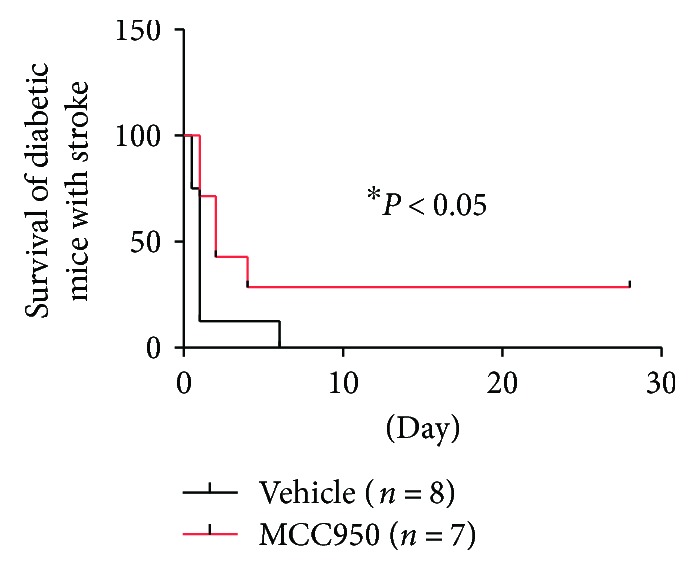
NLRP3 inhibition enhanced the 28-day survival rate in diabetic mice. The survival of diabetic mice was observed until twenty-eight days after middle cerebral artery occlusion. MCC950 enhanced the 28-day survival rate of cerebral ischemia-reperfusion injury in diabetic mice (^∗^*P* < 0.05). ^∗^*P* < 0.05 compared with the vehicle group.

To identify the role of NLRP3 inflammasome in diabetic mice with ischemia-reperfusion injury, we observed the 28-day survival rate. As shown in Figure 4, MCC950 significantly improved the 28-day survival rate of cerebral ischemia-reperfusion in diabetic mice (0.00 versus 28.571; *P* < 0.05 and *n* = 7 and 8).

### 3.4. NLRP3 Inhibition Had No Effect on the Infarction Size

**Figure 5:**
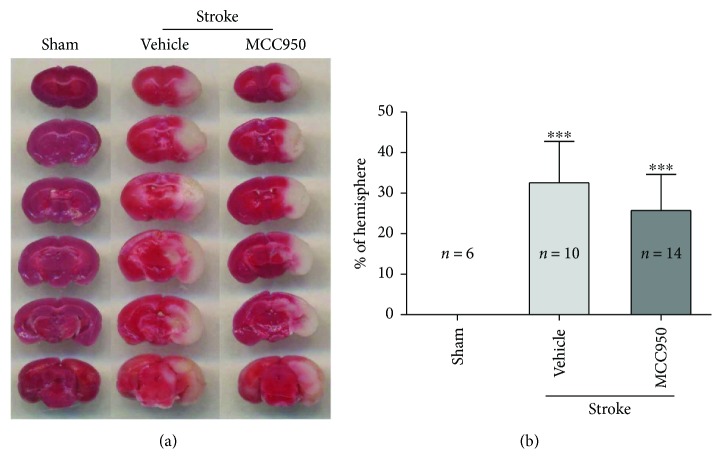
NLRP3 inhibition had no effect on the infarction size in diabetic mice after 24 hr MCAO. The left panel showed that representative 2% TTC-stained coronal sections indicated infarctions in the sham and stroke group. Quantification of the infarct volume was represented as a percent of the hemispheric volume 24 hr poststroke (shown in the right panel). Compared to the sham group, the stroke group showed conspicuous infarction volume percentage in diabetic mice (^∗∗∗^*P* < 0.001). Fifty milligrams per kilogram of MCC950 had no significant effect on the infarct size compared with the vehicle in diabetic mice (*P* > 0.05). ^∗∗∗^*P* < 0.001 compared with the sham group.

Compared to the sham group, the stroke group showed conspicuous infarction volume percentage in diabetic mice (0.000 ± 0.000 versus 32.565 ± 10.212; *P* < 0.001 and *n* = 6 and 10). However, intraperitoneal injection of MCC950 had no effect on the infarct size compared with the vehicle (25.784 ± 8.895 versus 32.565 ± 10.212, *P* > 0.05, *n* = 14 and 10) (Figure 5).

## 4. Discussion

Our study showed that the inhibition of NLRP3 inflammasome by MCC950 ameliorates cerebral ischemia-reperfusion injury in diabetic mice. Furthermore, the neurologic score was consistent with the mRNA transcription levels of NLRP3, IL-1*β*, and caspase-1 in the ischemia core. The diabetic mouse model with transient MCAO is well established with a neurobehavioral assessment in our study. Together, these findings demonstrated that MCC950, a small molecule of NLRP3 inhibitor, alleviates neurological deficits and improved long-term survival in diabetic mice, although there is no effect on the infarct size after brain ischemia reperfusion.

Ischemic stroke is one of the high-risk diseases threatening human health at present and is often associated with diabetes, which significantly aggravates the condition and prognosis [2, 14, 15]. There are evidences suggesting that effective regulation or inhibition of NLRP3 may help prevent or treat either ischemic stroke or diabetes when separated [16, 17]. However, there remain many questions, especially regarding cross-talk networks between NLRP3 inflammasome activation and the physiological course of diabetes concomitant with ischemic stroke. Therefore, it is important to explore the pathogenesis and figure out the effective prevention and treatment methods. Endogenous immune inflammatory reaction is a double-edged sword for the body. Although moderate inflammatory response is beneficial to the recovery of brain function after stroke, excessive and persistent inflammatory response is the main cause of tissue damage [18]. Cerebral ischemia and abnormal glucose level can both activate inflammasome and recruit simultaneously a variety of immune cells which mediate the activation of proinflammatory cytokine caspase-1 and IL-1*β*. Consistent with this, the mRNA expression levels of NLRP3, caspase-1, and IL-1*β* were significantly increased in our established diabetic MCAO mouse model.

In searching evidence of the essential role of NLRP3 inflammasome activation during ischemia-reperfusion injury, we hence employed a potent, selective, small-molecule inhibitor, MCC950. A study on MCC950 was published on Nature Medicine in 2015 [10], which has demonstrated that it may be a potential treatment for inflammation-related diseases *in vivo* and *in vitro.* Taking into account the influence factors of aging on stroke and diabetes, we included mice of the same age as our experiment object. As expected, our findings demonstrated that MCC950 improved symptoms of neurological function when administered before brain ischemia in a diabetic mice model. A recent study reported that MCC950 reduced dermal and airway inflammation and highlighted its pharmacologic properties in preclinical disease models [19]. Similar to their findings, our study also demonstrated that MCC950 effectively inhibits inflammation on cerebral ischemia under a diabetic environment. These effects may be related to the inhibition of the NF-*κ*B signaling pathway, decrease of the production of reactive oxygen species in mitochondria, and enhancement the autophagy function of cells [20–23]. With NLRP3 knockout mice, Yang's group found that NLRP3 deficiency reduced cerebral injury after ischemic stroke, including the reduction of cerebral infarction volume, cerebral edema, and brain barrier permeability [16]. In our study, MCC950 significantly improved the neurological function of diabetic mice; however, it did not reduce the area of cerebral infarction. This might due to the inhibitory effect of MCC950 which is not equivalent to that of NLRP3 knockout mice. As global knockout mice may have an impact on the development of ischemia and diabetes, our results may be more relevant on the clinical situation. In addition, it may be related to the ischemia and reperfusion time. It is noteworthy that MCC950 improved the long-term survival rate, combined with the neurobehavioral scores in diabetic mice with cerebral ischemia-reperfusion injury. We hypothesized that MCC950 may inhibit the systemic inflammatory cascade response after reperfusion, leading to a balance between anti-inflammatory and proinflammatory responses, not just in the cerebral infarction regions.

By inhibiting the activation of NLRP3 inflammasome, mRNA transcription of caspase-1 and IL-1*β* is also reduced. It is further suggested that the possible prognosis of ischemic stroke in diabetic mice may be due to the involvement of NLRP3 inflammasome. Previous studies [24] have pointed out that apoptosis within the ischemic penumbra may occur after several days, while necrosis starts in a few hours after the onset of acute brain ischemia in the ischemic core. Thus, we supposed that most of the neurons died 24 hours after stroke, and inflammasome was released from necrotic neurons in the core area of cerebral ischemia. Briefly, based on the animal study, we suggested that the NLRP3/IL-1*β* inflammasome pathway is involved in the severity of diabetic stroke and thereby resulting in caspase-1 activation and processing of cytoplasmic targets, including IL-1*β* and IL-18, and eventually leads to tissue damage. IL-1*β* is the most frequently studied member of the IL-1 family [25, 26]. The NLRP3 inflammasome has been correlated with obesity-induced insulin resistance and pancreas beta cell failure, which is the cause of type 2 diabetes [27]. Consistent with our finding, the use of MCC950 also inhibited the expression of IL-1*β* and caspase-1 in experimental NASH in mice [28]. It is suggested that the blocking efficacy of NLRP3 inflammasome may play a vital role in other chronic inflammatory diseases, because the proinflammatory factors in the downstream are inhibited and blocked.

In conclusion, our study indicated that when diabetic mice suffered from cerebral ischemia-reperfusion injury, it may be related to the sustained activation of NLRP3 inflammasome. MCC950, the small molecule of the NLRP3 specificity inhibitor, inhibits the activation of NLRP3 and downstream factors. As a result, it alleviated neurological deficits and improves survival outcomes. It is believed that NLRP3 inflammasome may become a potential drug target for the treatment of ischemic stroke combined with diabetes mellitus.

## Figures and Tables

**Table 1:** Sequences of primer used for real-time PCR assay (5′-3′).

Gene symbol	Gene description	Sequences of primer (mouse)	
NLRP3	NATCH-, LRR-, and PYD-containing protein 3	Forward	AGGAGGAAGAAGAAGAGAGGA
		Reverse	AGAGACCACGGCAGAAGC
IL-1*β*	Interleukin-1 beta	Forward	TTCAGGCAGGCAGTATCAC
		Reverse	CAGCAGGTTATCATCATCATCC
Casp-1	Caspase-1	Forward	CGTCTTGCCCTCATTATCTG
		Reverse	TCACCTCTTTCACCATCTCC
ACTB	*β*-Actin	Forward	GTGCTATGTTGCTCTAGACTTCG
		Reverse	ATGCCACAGGATTCCATACC
